# Supplementary material for: DutaFabs are engineered therapeutic Fab fragments that can bind two targets simultaneously
Source: Nat Commun. 2021 Jan 29;12:708. doi: 10.1038/s41467-021-20949-3 (PMC7846786; doi:10.1038/s41467-021-20949-3)
Supplement: Supplementary file 1 — Supplementary Information [file 41467_2021_20949_MOESM1_ESM.pdf]

Supplementary Table S1: Parameters of the crystal structures

Crystallographic data collection and refinement statistics

	Fab-VEGF Complex	FAB-PDGF Complex
Data collection		
Wavelength (Å)	1.0000	0.9760
Space group	$P2_1$	$P2_1$
Cell dimensions		
a, *b*, *c* (Å)	70.1, 107.9, 87.4	89.3, 75.5, 116.5
α, β, γ (deg)	90.0, 95.3, 90.0	90.0, 110.6, 90.0
Resolution (Å)	30.0-2.9 (3.1-2.9)[a]	30.0-3.0 (3.17-3.0) [a]
R_{merge} (%)	21.2 (84.2)	16.2 (77.3)
$I/\sigma I$	6.7 (1.5)	7.33 (1.83)
Completeness (%)	98.9 (98.2)	97.7 (92.8)
Redundancy	3.0 (3.0)	3.0 (2.96)
Refinement		
No. of reflections	28318 (4494)	29016 (4371)
No. of test set reflections (%)	1416 (5.0 %)	1451 (5.0 %)
R_{work}/R_{free}	21.2 / 28.0	25.3 / 30.4
No. of non-hydrogen atoms	8284	7935
Water	213	0
Average B-factor ($Å^2$)	37.8	62.9
R.m.s. deviations		
Bond lengths (Å)	0.007	0.012
Bond angles (deg)	1.17	1.68
Ramachandran plot quality		
Favoured regions	963 (94.1%)	922 (91.8%)
Favoured + allowed regions	1011 (98.8%)	1014 (99.0%)
Outliers	12 (1.2%)	10 (1.0%)

[a] Values in parentheses are for the highest resolution shell.

Supplementary Figure S1: DutaFab sequences and antigen contacts

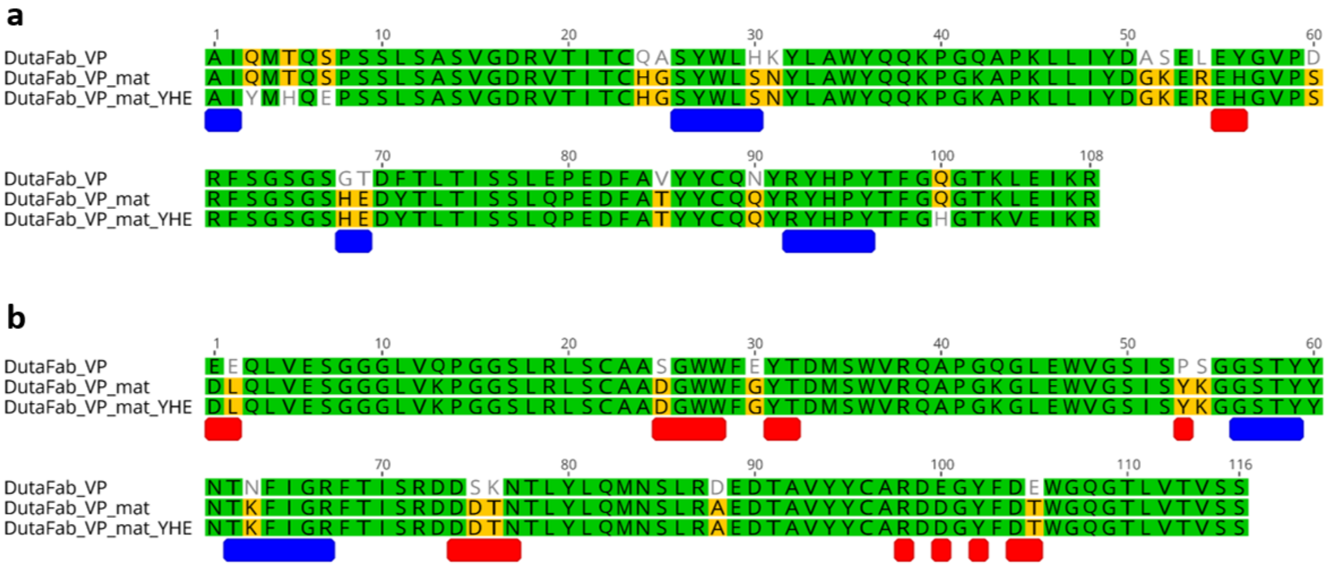

Supplementary Figure S1: DutaFab sequences and antigen contacts. (a) Alignment of Vk sequences, (b) alignment of VH sequences of DutaFab clones described in this study. Based on the X-ray structures of the DutaFab in complex with VEGF (PDB ID 6T9D) and in complex with PDGF (PDB ID 6T9E) residues that are closer than 5 Å to the respective targets are colour-coded in blue (VEGF paratope, L-side) and red (PDGF paratope, H-side), respectively.

Supplementary Figure S2: Humanness of DutaFabs

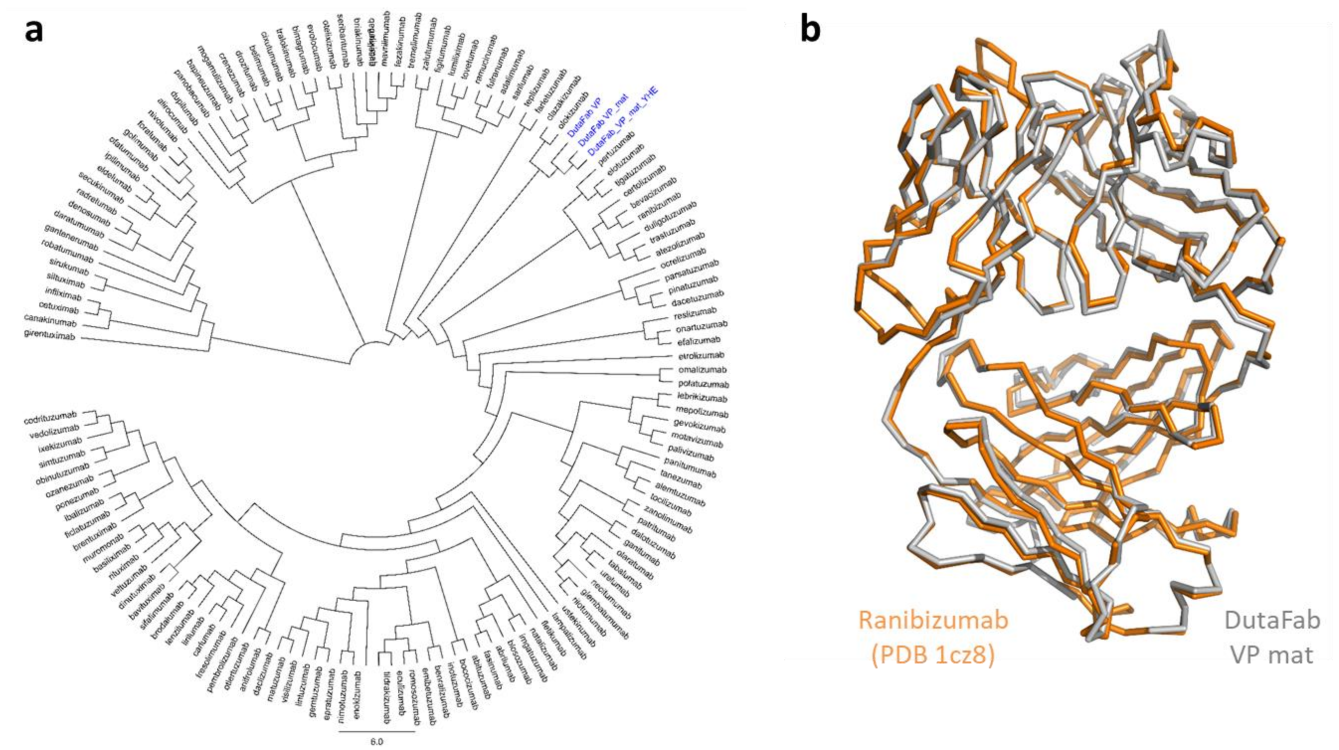

Supplementary Figure S2: Humanness of DutaFabs. (a) Sequence homology to INN-set: phylogenetic tree showing high sequence relationship to conventional Fabs. (b) Comparison of the structures of ranibizumab (orange) and DutaFab VP mat (grey). Fab structures were superimposed using C-alpha atoms.

Supplementary Table S2: Cross-reactivities of antibodies used for the in-vivo study

Affinities were determined by SPR with the binding partners mentioned in the table header and expressed as K_D (nM).

Antibody	human VEGF-A121	rat VEGF-A121
**DutaFab VP mat YHE**	0.005	0.043
**B20.4.1**	2.2	3.5

Recombinant rat PDGF-BB was not available in sufficient purity to allow for exact affinity determination. Only qualitative SPR binding data could be obtained which indicate cross-reactivity of DutaFab VP mat with rat PDGF-BB.

Supplementary Figure S3: Affinity analysis of PDGF-BB to DutaFab VP mat YHE via Kinetic Exclusion Assay (KinExA)

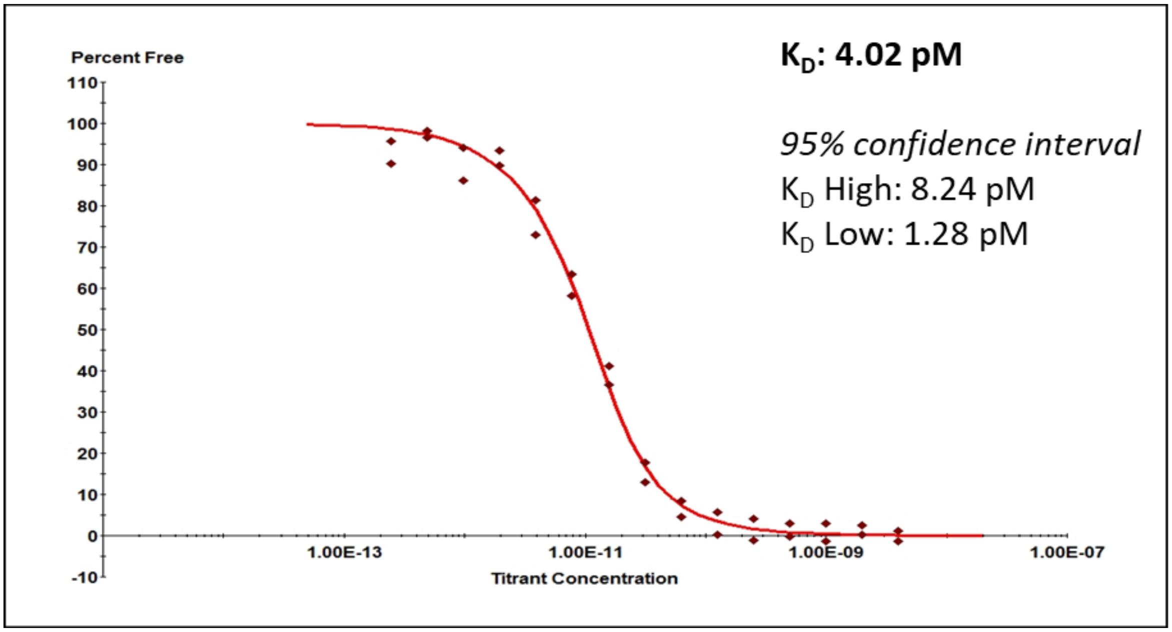

The affinity was determined by Kinetic Exclusion Assay (KinExA), using 50 pM DutaFab and a duplicate titration of 244 fM to 4 nM of PDGF-BB.

Supplementary Figure S4: Affinity analysis of VEGF A-121 to DutaFab VP mat YHE via Kinetic Exclusion Assay (KinExA)

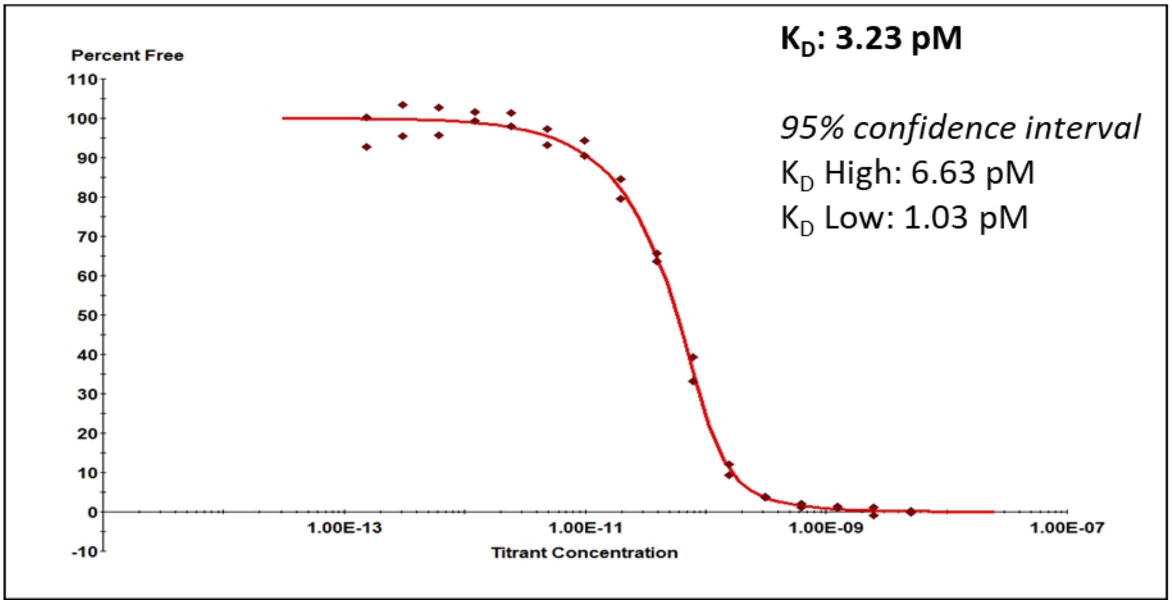

The affinity was determined by Kinetic Exclusion Assay (KinExA), using 100 pM DutaFab and a duplicate titration of 122 fM to 2 nM VEGFA-121.

Supplementary Table S3: Independent binding of VEGF and PDGF to DutaFab VP_mat

SPR signal at 460 seconds (=end of association period) of the SPR curves shown in Figure 4d. The signal height reached with DutaFab pre-incubated with a VEGF+PDGF-BB mix is set to 100 %.

**Inject 1**	**Inject 2**	**Signal [%]**
VEGFA-121	PDGF-BB	98
PDGF-BB	VEGFA-121	98
Mix (VEGF + PDGF-BB)	HBS-P	100

Supplementary Table S4: Polyreactivity assay

Polyreactivity was assessed with an ELISA-based assay. ELISA plates were coated with the reagents mentioned in the table header. These reagents are intended to span a wide range of abundant biomolecules. Binding of DutaFabs was measured with chemoluminescence and compared to a known polyreactive antibody (clone L31D11, Schanzer et al., https://dx.doi.org/10.1080%2F19420862.2016.1160989).

Sample	PDGF-BB	Eph A4-ECD	His-tone H4	Car-dio-lipin	He-parin	hu CD40	Para-thyroid hor-mone	DNA	Apo-lipo-pro-tein A-1	Hemo-cyanin	Stre-pta-vidin	HSP 70	In-sulin	E.coli Ly-sate	Gel-atine 2%	PBS/ BSA Block
**DutaFab VP mat**	1.97	-0.01	-0.01	-0.01	-0.03	-0.03	-0.02	-0.03	-0.02	-0.01	-0.01	-0.01	-0.01	0.00	0.00	0.01
**DutaFab VP mat YHE**	1.95	0.00	-0.01	0.00	-0.01	0.00	0.00	-0.01	0.00	0.00	0.01	-0.01	0.00	0.01	0.00	0.00
**pos. control anti-IGF-1R clone L31D11**	2.39	0.64	0.47	0.75	0.64	0.67	0.88	2.51	1.35	1.71	0.75	1.61	2.05	2.52	0.22	0.77
