# Supplementary material for: DutaFabs are engineered therapeutic Fab fragments that can bind two targets simultaneously
Source: Nat Commun. 2021 Jan 29;12:708. doi: 10.1038/s41467-021-20949-3 (PMC7846786; doi:10.1038/s41467-021-20949-3)
Supplement: Supplementary file 2 — Reporting Summary [file 41467_2021_20949_MOESM2_ESM.pdf]

nature research

Corresponding author(s): Hubert Kettenberger, Roland Beckmann

Last updated by author(s): Dec 10, 2020

Reporting Summary

Nature Research wishes to improve the reproducibility of the work that we publish. This form provides structure for consistency and transparency in reporting. For further information on Nature Research policies, see our Editorial Policies and the Editorial Policy Checklist.

Statistics

For all statistical analyses, confirm that the following items are present in the figure legend, table legend, main text, or Methods section.

n/a	Confirmed	
☐	☒	The exact sample size (*n*) for each experimental group/condition, given as a discrete number and unit of measurement
☐	☒	A statement on whether measurements were taken from distinct samples or whether the same sample was measured repeatedly
☐	☒	The statistical test(s) used AND whether they are one- or two-sided<br>*Only common tests should be described solely by name; describe more complex techniques in the Methods section.*
☒	☐	A description of all covariates tested
☒	☐	A description of any assumptions or corrections, such as tests of normality and adjustment for multiple comparisons
☐	☒	A full description of the statistical parameters including central tendency (e.g. means) or other basic estimates (e.g. regression coefficient) AND variation (e.g. standard deviation) or associated estimates of uncertainty (e.g. confidence intervals)
☐	☒	For null hypothesis testing, the test statistic (e.g. *F*, *t*, *r*) with confidence intervals, effect sizes, degrees of freedom and *P* value noted<br>*Give P values as exact values whenever suitable.*
☒	☐	For Bayesian analysis, information on the choice of priors and Markov chain Monte Carlo settings
☒	☐	For hierarchical and complex designs, identification of the appropriate level for tests and full reporting of outcomes
☒	☐	Estimates of effect sizes (e.g. Cohen's *d*, Pearson's *r*), indicating how they were calculated

Our web collection on statistics for biologists contains articles on many of the points above.

Software and code

Policy information about availability of computer code

Data collection	Xray data: XDS; Calorimetry da ta: MicroCal; OD450 ELISA readouts: iControl (Tecan); SPR data: Biacore; Angiography data: Heidelberg Retinal; DLS data (viscosity): Dynamics V7 (Wyatt); HPLC-data: Chromeleon 7; thermal unfolding data: Optim1000; Kinetic Exclusion assay: KinExA Pro
Data analysis	Xray data processing: CCP4, Phaser, Refmac5, COOT; protein structure data visualization: PyMol 2.3.1; calorimetry data: Origin 7; curve fitting (Fig. 3+5) and statistical analysis in Fig. 6, visualization of Fig. 7: GraphPad Prism V7.04; SPR data: Biacore; Kinetic Exclusion assay: KinExA Pro; ELISA data handling: MS Excel 2016; phylogenetic tree (Fig. S2a: Geneious Prime)

For manuscripts utilizing custom algorithms or software that are central to the research but not yet described in published literature, software must be made available to editors and reviewers. We strongly encourage code deposition in a community repository (e.g. GitHub). See the Nature Research guidelines for submitting code & software for further information.

Data

Policy information about availability of data

All manuscripts must include a data availability statement. This statement should provide the following information, where applicable:

- Accession codes, unique identifiers, or web links for publicly available datasets
- A list of figures that have associated raw data
- A description of any restrictions on data availability

Xray crystallography data that support the findings of this study have been deposited in the Protein Database (www.rcsb.org) with the accession codes 6T9D and 6T9E, respectively. Calorimetry raw data shown in Figure 2a, SPR data shown in Figure 4d, stability and viscosity data shown in Fig. 7, and Kinexa binding instrument readouts shown in Figure S3 and S4 are available from the corresponding author upon reasonable request. The authors declare that all other data supporting the findings of this study are available within the paper and its supplementary information files.

Field-specific reporting

Please select the one below that is the best fit for your research. If you are not sure, read the appropriate sections before making your selection.

- [x] Life sciences
- [ ] Behavioural & social sciences
- [ ] Ecological, evolutionary & environmental sciences

For a reference copy of the document with all sections, see nature.com/documents/nr-reporting-summary-flat.pdf

Life sciences study design

All studies must disclose on these points even when the disclosure is negative.

Sample size	For the in vivo efficacy study, 14 eyes were used for the bispecific anti-VEGF/anti-PDGF treatment based on investigator experience of the usual variability of angiography studies. For the negative isotype control, half the number (i.e. 7 eyes) as for the bispecific compound were used, to minimize the number of animals receiving negative control treatment for ethical reasons. For the positive anti-VEGF monotherapy control, an additional 2 eyes were included (i.e. a total of 16 eyes was used), to minimize the risk of drop-outs and thus not obtaining a significant read-out for the important positive control, in case of unforeseen adverse events in any of the study animals.<br>For biochemical ELISAs (Figure 3) a sample size of n=2 was utilized, as these are stable assays with 100s of plates being processed in the authors' laboratories each month. For the baseline assay (Figure 5b) a sample size of n=3 was chosen, as this is a new assay format specially developed for the present study. For cellular assays (Figure 5c) a sample size of n=3 was chosen, as live cells may show somewhat higher variability compared to a biochemical ELISA formats.
Data exclusions	For evaluation of fluorescence angiography data, the following procedure was used: If the two scores assigned to a lesion do not match, the higher score will be used for analysis. This analysis is a qualitative analysis. If an anomaly of an eye is observed and the angiography is not evaluable, fluorescence angiography data were not evaluated. These exclusion criteria were pre-established.
Replication	All the DutaFab clones shown in the present study are part of much larger groups, of initial binders, affinity-matured variants, and final engineered variants (with cooperativity motif). Thus all clones were analysed many times in multiple contexts, in comparison to various other DutaFab clones before and after affinity maturation. Thus for most experiments we have a much larger body of data, where all the findings of this manuscript were confirmed multiple times, even when it was not necessarily in the same plate layout as the one presented in this manuscript, thus giving the authors confidence in the findings. Exceptions to this are the crystallography work and the in vivo study, both of which were only performed once with the most potent VEEGF-PDGF DutaFab clone. In the case of Xray crystallography this is the unusual approach in the industry; in the case of the animal study this approach is necessary for ethical reasons.
Randomization	For the in-vivo data shown in Fig. 6, animals with no visible defect were randomly assigned to the study groups. For the other experiments, randomization was not relevant.
Blinding	The in-vivo data shown in Fig. 6 (leakage of fluorescein) was evaluated in the angiograms by two masked examiners to the study groups. For other experiments, blinding was not relevant because experimental readouts such as absorbance, fluorescence, etc. are generated by the respective devices without operator bias.

Reporting for specific materials, systems and methods

We require information from authors about some types of materials, experimental systems and methods used in many studies. Here, indicate whether each material, system or method listed is relevant to your study. If you are not sure if a list item applies to your research, read the appropriate section before selecting a response.

Materials & experimental systems

n/a	Involved in the study	
[ ]	[x]	Antibodies
[ ]	[x]	Eukaryotic cell lines
[x]	[ ]	Palaeontology and archaeology
[ ]	[x]	Animals and other organisms
[x]	[ ]	Human research participants
[x]	[ ]	Clinical data
[x]	[ ]	Dual use research of concern

Methods

n/a	Involved in the study	
[x]	[ ]	ChIP-seq
[x]	[ ]	Flow cytometry
[x]	[ ]	MRI-based neuroimaging

Antibodies

Antibodies used	DutaFabs (sequence disclosed in this study); aflibercept and brolucizumab (produced in-house in HEK293 transient expression according to the published INN sequences; anti-DIG Fab and anti-VEGFA B20.4.1 (produced in-house in HEK293 transient expression); anti-T7 antibody (Merck catalogue number 69968), polyclonal anti-Fab antibody (Sigma catalogue no. I5260), anti-VEGFA (R&D, BAF293), or anti-PDGF-BB antibody (R&D; BAF220)
Validation	anti-DIG Fab (Metz, S. et al. Bispecific digoxigenin-binding antibodies for targeted payload delivery. Proceedings of the National

	Academy of Sciences of the United States of America 108, 8194–9 (2011).); anti-VEGFA B20.4.1 (Liang, W.-C. et al. Cross-species vascular endothelial growth factor (VEGF)-blocking antibodies completely inhibit the growth of human tumor xenografts and measure the contribution of stromal VEGF. The Journal of biological chemistry 281, 951–61 (2006).) commercial antibodies (anti-T7, polyclonal anti-Fab, anti-VEGFA, anti-PDGF-BB) were used without further validation.

Eukaryotic cell lines

Policy information about cell lines

Cell line source(s)	HEK Expi293 cells (ThermoFisher); GloResponse™ NFAT-RE-luc2P HEK293 cells (Promega); human umbilical vein/vascular endothelial cells (HUVEC) (ATCC)
Authentication	Cell lines were purchased at the respective vendors and used without further authentication.
Mycoplasma contamination	All cell lines tested negatively for mycoplasma.
Commonly misidentified lines (See ICLAC register)	No commonly misidentified cell lines were used in this study.

Animals and other organisms

Policy information about studies involving animals; ARRIVE guidelines recommended for reporting animal research

Laboratory animals	Pigmented Brown Norway rats (Rattus norvegicus), which were males and 8-10 weeks old at the time of induction. Housing conditions are described in the manuscript.
Wild animals	The study did not involve wild animals.
Field-collected samples	The study did not involve samples collected from the field.
Ethics oversight	All standard operating procedures and protocols described in this manuscript have been reviewed by Iris Pharma Internal Ethics Committee. All animals were treated according to the Directive 2010/63/UE European Convention for the Protection of Vertebrate Animals used for Experimental and Other Scientific Purposes and to the Association for Research in Vision and Ophthalmology (ARVO) Statement for the Use of Animals in Ophthalmic and Vision Research. This information has also been added to the manuscript.

Note that full information on the approval of the study protocol must also be provided in the manuscript.
