# Supplementary material for: DutaFabs are engineered therapeutic Fab fragments that can bind two targets simultaneously
Source: Nat Commun. 2021 Jan 29;12:708. doi: 10.1038/s41467-021-20949-3 (PMC7846786; doi:10.1038/s41467-021-20949-3)
Supplement: Supplementary file 4 — Source Data [file 41467_2021_20949_MOESM4_ESM.pdf]

Reviewer #1 (Remarks to the Author):

Manuscript NCOMMS 252434_0 describes a new bispecific antibody platform (DutaFab) that is based on evolving and combining two structurally independent paratopes in a single Fv. Unlike previous technologies that combined single domain VL and VH paratopes to bispecific paratopes in a single Fv, each paratope of the DutaFabs involves VL and VH components, i.e. HCDR1, HCDR3, and LCDR2 for the "H site paratope" and LCDR1, LCDR3, and HCDR2 for the "L site paratope". The design of the scaffold and libraries is rigorous. The novel platform is applied to the de novo generation (followed by tailored affinity maturation) of a bispecific antibody in Fab format that simultaneously binds VEGF-A and PDGF-BB with picomolar affinity and as such revealed excellent biophysical properties and preclinical in vitro and in vivo activity. X-ray crystallography structures of the bispecific antibody in complex with either VEGF-A or PDGF-BB (unfortunately not both) further support the ingenuity of this design. Overall, this is an outstanding manuscript of broad interest in the field of therapeutic antibodies. A few minor points:
1) As broad utility is important for any new platform, can the authors elaborate on additional targets they have successfully combined as DutaFabs, in particular membrane targets?
2) Is the DutaFab that "has recently succeeded in entering a clinical trial" VP mat YHE or a different one?
3) Clarify in which format (Fab or IgG), the negative and positive control antibodies (anti-Dig and anti-VEGFA B20.4.1) were used in the in vivo experiment shown in Fig. 6. Confirm the cross-reactivity of DutaFab with rat VEGFA and PDGF-BB, and the cross-reactivity of B20.4.1 with rat VEGFA. Compared to B20.4.1, is the better performance of the DutaFab due to its ability to simultaneously block VEGFA and PDGF-BB or due to its ability to cooperatively block the VEGFA and/or PDGF-BB dimer?
4) The authors provide strong evidence that the independent paratopes provide sufficient interaction areas to bind targets with high affinity. If they have any data revealing equally high specificity as conventional antibodies, it would further strengthen their conclusions.
5) Should the second paragraph of the chapter "Diversification and library construction" start with "As illustrated in Figure 1b" (rather than 2c)?
6) In Figure 2c, S (for stability) and C (conformation) are easy to confuse with the single letter amino acid codes of serine and cysteine – use Greek letters or numbers instead?
7) References 6-8 and 35 are incomplete. Reference 44 is missing.

Reviewer #2 (Remarks to the Author):

The authors describe a novel bispecific antibody format (DutaFab) and demonstrate simultaneous binding to VEGFA and PDGF-BB. This technology is distinct from dual action Fabs previously described by others (Bostrom et al.) and has some potential advantages. This work is likely of broad interest to antibody engineers and antibody drug developers.

Several areas of this manuscript require attention:

[1] The title and abstract emphasize 'high affinity' antigen binding. An SPR sensorgram is provided to convincingly simultaneous binding to both VEGFA and PDGF-BB. Surprisingly, however, Kd values are not reported, only IC50 values. Please provide Kd values, preferably at physiologic temperature, for VEGFA and PDGF-BB to support the high affinity claim: i) VEGFA alone, ii) PDGF-BB alone, iii) Prebind PDGF-BB then bind VEGFA, iv) Prebind VEGFA then bind PDGF-BB. i.e., evaluate whether binding of

one antigen influences the binding affinity for the second antigen.

[2] References #1-3 are cited to support the broad statement:
“As monoclonal antibodies continue to play a central role in the development of new large molecule therapeutics, an increasing number of bispecific antibody approaches has been developed and described in the literature.”
Citation of recent review articles such as Ref. 1 seem appropriate to this reviewer, whereas citations of older primary publications seem much less relevant to support this broad statement.

[3] Results, second paragraph. I assume that the statements on L and H side paratopes are based upon the homology models. If so, please state this more directly and clearly.

[4] Page 5. The reported Tm values for some of the DutaFabs are astonishingly high (<100 ˚C). This is an interesting and potentially important finding that warrants a more complete description of how it was accomplished than ‘… combining the stabilizing features resulted in extremely thermostable DutaFabs .’

[5] ‘Very high affinities’ is vague and subjective. Please provide something specific, e.g., Kd value <100 pM (say).

[6] Discussion. There is a theoretical risk that binding of the DutaFab to antigen A may sterically block subsequent binding to antigen B depending upon the antigens and the epitopes. Some discussion is required of the success rate in generating DutaFabs. Specifically, how many antigen pairs and DutaFabs have been evaluated? What proportion has the property that binding to antigen A does not interfere with binding to antigen B (and vice versa)?

[7] Discussion, closing statement. Please provide the clinical trial number for the anti-VEGF-A/PDGF-BB DutaFab clinical trial.

Reviewer #3 (Remarks to the Author):

The authors present a compelling case for the design and characterisation of a dual specific antibody that could be effectively used to develop a new method for clinical intervention in diseases like age-related macular degeneration. The approach is highly innovative and the presented data is compelling to me as a non-expert.

While I fully appreciate the technical know-how and the novelty, it is not clear to me how DuaFab compounds will revolutionising ophthalmology maybe because this is not as clearly defined for a non-expert like me. This should require some consideration. While the DuaFab will recognise both VEGF and PDGF, it would only bind to both if they are in close proximity. If this is a likely scenario in AMD, this has to be elaborated on.

Overall the text is written clearly, though very technical, naturally. Still, it is easy to follow structure and logic. There are a few small issues that should also be attended to including:
1) no definition what MOAs are
2) would heterodimericFv be heterodimeric Fv?

3) while I believe this is real word worlds I m not very keen on these: developability, concentrability etc.

POINT-BY-POINT RESPONSES TO REFEREES' COMMENTS

Reviewer #1 (Remarks to the Author):

Manuscript NCOMMS 252434_0 describes a new bispecific antibody platform (DutaFab) that is based on evolving and combining two structurally independent paratopes in a single Fv. Unlike previous technologies that combined single domain VL and VH paratopes to bispecific paratopes in a single Fv, each paratope of the DutaFabs involves VL and VH components, i.e. HCDR1, HCDR3, and LCDR2 for the "H site paratope" and LCDR1, LCDR3, and HCDR2 for the "L site paratope". The design of the scaffold and libraries is rigorous. The novel platform is applied to the de novo generation (followed by tailored affinity maturation) of a bispecific antibody in Fab format that simultaneously binds VEGF-A and PDGF-BB with picomolar affinity and as such revealed excellent biophysical properties and preclinical in vitro and in vivo activity. X-ray crystallography structures of the bispecific antibody in complex with either VEGF-A or PDGF-BB (unfortunately not both) further support the ingenuity of this design. Overall, this is an outstanding manuscript of broad interest in the field of therapeutic antibodies. A few minor points:*

1) As broad utility is important for any new platform, can the authors elaborate on additional targets they have successfully combined as DutaFabs, in particular membrane targets?

We agree with the reviewer that demonstration of the broad utility of a new molecule platform is important. To address this concern, we have now stated the fact that eight membrane proteins have been targeted in Results section "Panning, screening and success rate", and we have provided more granularity and detailed numbers in the Results section "Generation of bispecific DutaFabs".

2) Is the DutaFab that "has recently succeeded in entering a clinical trial" VP mat YHE or a different one?

The VEGF-PDGF-binding DutaFab described in this manuscript is currently not being studied in clinical trials. Nonetheless, a different DutaFab, based on the same molecular architecture, is being investigated for neovascular AMD. We have added a sentence at the end of the discussion section and provided the clinical trial number (NCT04567303).

3) Clarify in which format (Fab or IgG), the negative and positive control antibodies (anti-Dig and anti-VEGFA B20.4.1) were used in the in vivo experiment shown in Fig. 6.

We confirm that all 3 antibodies in the study were used in the Fab fragment format, and the sample description in the Results section ("In vivo efficacy of the VEGF-PDGF DutaFab" has been amended accordingly. Further, a Table S2 has been added to the supplement detailing the affinities of the antibodies measured against rat VEGF.

Confirm the cross-reactivity of DutaFab with rat VEGFA and PDGF-BB, and the cross-reactivity of B20.4.1 with rat VEGFA. Compared to B20.4.1, is the better performance of the DutaFab due to its ability to simultaneously block VEGFA and

PDGF-BB or due to its ability to cooperatively block the VEGFA and/or PDGF-BB dimer?

The reviewer raises an important question as to whether the better performance of the DutaFab is indeed due to the dual biology of neutralizing both VEGF and PDGF, or whether it might be due to better performance on the VEGF target alone. The positive control Fab B20.4.1 was chosen because of its verified and published good efficacy in rodent CNV models. Importantly, rats have a vitreous volume of up to approximately 14μL, and the antibody dose of 10μg per eye actually results in a very high vitreal concentration of approximately 14μM for the different Fab fragments (which have a molecular weight of 50 kDa). For the positive control Fab B20.4.1, this concentration is approximately 4000-fold above its K_D for rat VEGF, resulting in full neutralization of the rat VEGF present in the model, and we have now added an explanation in the Online Methods section to clarify this for the reader. Given the full neutralization of rat VEGF by the positive control, we suggest it is reasonable to preliminarily attribute the superior performance of the DutaFab to the additional neutralization of rat PDGF; nonetheless, we also fully agree with the reviewer that the interpretation of such *in vivo* data should be approached with caution, and have accordingly added a statement in Results section “In vivo efficacy of the VEGF-PDGF DutaFab”, that an effect of the DutaFab’s high potency towards VEGF and/or an effect of its high folding stability cannot be excluded. In any case, we would like to reiterate that the presented *in vivo* data serve their main purpose, not of exploring the dual biology of VEGF and PDGF inhibition, but of demonstrating in vivo stability and bioavailability of the novel DutaFab format.

4) The authors provide strong evidence that the independent paratopes provide sufficient interaction areas to bind targets with high affinity. If they have any data revealing equally high specificity as conventional antibodies, it would further strengthen their conclusions.

We agree with the reviewer that specificity is an important feature for any potential biotherapeutic. We have therefore added Supplementary Table S4, showing data that confirm a negative polyreactivity readout for the affinity matured DutaFab VP_mat and the final DutaFab clone VP_mat_YHE. We have added a corresponding sentence at the end of Results section “Further VEGF potency improvement based on structure”.

5) Should the second paragraph of the chapter “Diversification and library construction” start with “As illustrated in Figure 1b” (rather than 2c)?

We have modified the sentence to read “As illustrated in Figure 1b and further detailed in Figure 2c”.

6) In Figure 2c, S (for stability) and C (conformation) are easy to confuse with the single letter amino acid codes of serine and cysteine – use Greek letters or numbers instead?

We have modified Figure 2c to make this point clear for the reader, using # to indicate residues critical for stability and $ to indicate residues critical for conformation.

7) References 6-8 and 35 are incomplete. Reference 44 is missing.

We have corrected this issue.

*Obtaining the co-crystal structure of a DutaFab in complex with both ligands was unsuccessful, likely because both ligands are homodimers. Mixing them with a bispecific, bivalent DutaFab at concentrations required for crystallization led - as expected - to aggregation/immune complex formation.

Reviewer #2 (Remarks to the Author):

The authors describe a novel bispecific antibody format (DutaFab) and demonstrate simultaneous binding to VEGFA and PDGF-BB. This technology is distinct from dual action Fabs previously described by others (Bostrom et al.) and has some potential advantages. This work is likely of broad interest to antibody engineers and antibody drug developers.

Several areas of this manuscript require attention:

[1] The title and abstract emphasize 'high affinity' antigen binding. An SPR sensorgram is provided to convincingly simultaneous binding to both VEGFA and PDGF-BB. Surprisingly, however, Kd values are not reported, only IC50 values. Please provide Kd values, preferably at physiologic temperature, for VEGFA and PDGF-BB to support the high affinity claim: i) VEGFA alone, ii) PDGF-BB alone, iii) Prebind PDGF-BB then bind VEGFA, iv) Prebind VEGFA then bind PDGF-BB. i.e., evaluate whether binding of one antigen influences the binding affinity for the second antigen.

Supplementary figures S3 and S4, as well as Supplementary table S3, were added to provide the requested data supporting the high affinity claim. Both the VEGF-A and PDGF-BB targets are dimeric and can result in potential avidity effects when affinities are measured by SPR; moreover, the PDGF-BB dimer has a very high pI of approximately 11.5, and its strong positive charge can cause artefacts when using SPR. Hence we chose KinExA to determine affinities, and while KinExA is not operated at physiologic temperature, it provides accurate values at 25°C determined entirely in solution without the above pitfalls of SPR. We hope the reviewer agrees to this approach, given the nature of the targets in this particular study. The K_D values for both VEGF-A (3 pM) and PDGF-BB (4 pM) further support the high affinities already implied by the IC_{50} data. We have added two sentences in the Results section "Further VEGF potency improvement based on structure", and experimental details of the KinExA work in the online methods. Concomitant binding of VEGF and PDGF, i.e. the fact that both ligands can be bound simultaneously and do not interfere with kinetics of binding has been shown in Figure 4d. The fact that pre-binding of VEGF or PDGF leads to the same signal height as pre-binding to both of the ligands before capturing the complexes on the SPR chip surface is seen as a proof of this fact.

[2] References #1-3 are cited to support the broad statement:

"As monoclonal antibodies continue to play a central role in the development of new large molecule therapeutics, an increasing number of bispecific antibody approaches has been developed and described in the literature."

Citation of recent review articles such as Ref. 1 seem appropriate to this reviewer, whereas citations of older primary publications seem much less relevant to support this broad statement.

We agree with the reviewer and have changed the citation to include only reference #1.

[3] Results, second paragraph. I assume that the statements on L and H side paratopes are based upon the homology models. If so, please state this more directly and clearly.

The reviewer's assumption is correct, and we have re-structured the first three sentences of the second paragraph of the Results section, to make this clear for the reader.

[4] Page 5. The reported Tm values for some of the DutaFabs are astonishingly high (<100 ˚C). This is an interesting and potentially important finding that warrants a more complete description of how it was accomplished than '… combining the stabilizing features resulted in extremely thermostable DutaFabs .'

We agree with the reviewer that more detail may be of interest to the reader, and have modified the section to explain that conserving interface CDR residues VH35, VH50, VK34 and VK91 as the invariant residues shown in Figure 2c produced the highest thermostabilities.

[5] 'Very high affinities' is vague and subjective. Please provide something specific, e.g., Kd value <100 pM (say).

We have changed the first sentence of the second paragraph of Results section "DutaFab against VEGFA and PDGF-BB" to state clearly that low pM affinities were the project goal. We have also added Supplementary figures S3 and S4, showing affinity measurements by Kinetic Exclusion Assay (KinExA). The measured KD values for both VEGF-A (3 pM) and PDGF-BB (6 pM) further support the high affinities already implied by the IC_{50} data. We have added two sentences in Results section "Further VEGF potency improvement based on structure", and experimental details of the KinExA work in the online methods.

[6] Discussion. There is a theoretical risk that binding of the DutaFab to antigen A may sterically block subsequent binding to antigen B depending upon the antigens and the epitopes. Some discussion is required of the success rate in generating DutaFabs. Specifically, how many antigen pairs and DutaFabs have been evaluated? What proportion has the property that binding to antigen A does not interfere with binding to antigen B (and vice versa)?

We agree with the reviewer that the success rate in generating DutaFabs can be explained more clearly, and we have now provided more granularity and detailed numbers in the Results section "Generation of bispecific DutaFabs". We also agree with the reviewer that there is the "risk" that binding of the DutaFab to antigen A may sterically block subsequent binding to antigen B, and also that the factors determining this simultaneous or mutually exclusive binding mode are the nature and size of the targets, as well as epitope. However, we view this not so much as a risk potentially leading to project failure, but rather as an

opportunity for projects that would benefit from mutually exclusive binding, and indeed, we have performed several projects of both types (additive and mutually exclusive binding mode). While we feel that the current report should explain the new platform with only one detailed example, for which we have chosen the additive binding scenario, we have now added an explanation regarding the existence of both options in the Discussion (please see new 4th paragraph).

[7] Discussion, closing statement. Please provide the clinical trial number for the anti-VEGF-A/PDGF-BB DutaFab clinical trial.

The VEGF-PDGF-binding DutaFab described in this manuscript is currently not being studied in clinical trials. Nonetheless, a different DutaFab, based on the same molecular architecture, is being investigated for neovascular AMD. We have added a sentence at the end of the discussion section and provided the clinical trial number (NCT04567303).

Reviewer #3 (Remarks to the Author):

The authors present a compelling case for the design and characterisation of a dual specific antibody that could be effectively used to develop a new method for clinical intervention in diseases like age-related macular degeneration. The approach is highly innovative and the presented data is compelling to me as a non-expert.

While I fully appreciate the technical know-how and the novelty, it is not clear to me how DuaFab compounds will revolutionising ophthalmology maybe because this is not as clearly defined for a non-expert like me. This should require some consideration. While the DuaFab will recognise both VEGF and PDGF, it would only bind to both if they are in close proximity. If this is a likely scenario in AMD, this has to be elaborated on.*

Overall the text is written clearly, though very technical, naturally. Still, it is easy to follow structure and logic. There are a few small issues that should also be attended to including:

1) no definition what MOAs are

We have changed the heading of the first paragraph of the Discussion to spell out the term “Mechanisms of Action”, to rectify this unclarity.

2) would heterodimericFv be heterodimeric Fv?

Yes, we corrected this spelling mistake.

3) while I believe this is real word worlds I m not very keen on these: developability, concentrability

In order to improve clarity for the broad readership of the journal, we have changed the term “concentratability” to “solubility”, in Results section “Developability of VEGF-PDGF DutaFab”. However, we would like to keep the term “Developability”, as it is now a widely used term in this field as seen by approximately 300 search results in PubMed and approximately 17’000 hits in google scholar.

* The two targets of the DutaFab presented in detail in this study, VEGF and PDGF, are soluble proteins (not membrane-anchored proteins) which play a role in diseases such as AMD. Hence, close proximity and concomitant binding can occur in solution *in-vivo.*

Reviewer #1 (Remarks to the Author):

The authors have thoroughly addressed my critique. The added text and data further strengthen the manuscript.

Christoph Rader

Reviewer #2 (Remarks to the Author):

The authors have provided a robust point-by-point response to all of the reviews. Their manuscript is much stronger as a result.
